# Machine Learning Algorithms for the Prediction of Central Lymph Node Metastasis in Patients With Papillary Thyroid Cancer

**DOI:** 10.3389/fendo.2020.577537

**Published:** 2020-10-21

**Authors:** Yijun Wu, Ke Rao, Jianghao Liu, Chang Han, Liang Gong, Yuming Chong, Ziwen Liu, Xiequn Xu

**Affiliations:** ^1^Department of General Surgery, Peking Union Medical College Hospital, Chinese Academy of Medical Sciences and Peking Union Medical College, Beijing, China; ^2^Peking Union Medical College, Chinese Academy of Medical Sciences, Beijing, China

**Keywords:** machine learning, cross-validation, central lymph node metastasis, papillary thyroid cancer, feature selection

## Abstract

**Background:**

Central lymph node metastasis (CLNM) occurs frequently in patients with papillary thyroid cancer (PTC), but performing prophylactic central lymph node dissection is still controversial. There are no reliable models for predicting CLNM. This study aimed to develop predictive models for CLNM by machine learning (ML) algorithms.

**Methods:**

Patients with PTC who underwent initial thyroid resection at our hospital between January 2018 and December 2019 were enrolled. A total of 22 variables, including clinical characteristics and ultrasonography (US) features, were used for conventional univariate and multivariate analysis and to construct ML-based models. A 5-fold cross validation strategy was used for validation and a feature selection approach was applied to identify risk factors.

**Results:**

The areas under the receiver operating characteristic curve (AUC) of 7 models ranged from 0.680 to 0.731. All models performed significantly better than US (AUC=0.623) in predicting CLNM (P<0.05). In decision curve, most of the models also performed better than US. The gradient boosting decision tree model with 7 variables was identified as the best model because of its best performance in both ROC (AUC=0.731) and decision curves. Based on multivariate analysis and feature selection, young age, male sex, low serum thyroid peroxidase antibody and US features such as suspected lymph nodes, microcalcification and tumor size > 1.1 cm were the most contributing predictors for CLNM.

**Conclusions:**

It is feasible to develop predictive models of CLNM in PTC patients by incorporating clinical characteristics and US features. The ML algorithm may be a useful tool for the prediction of lymph node metastasis in thyroid cancer.

## Introduction

Thyroid cancer is the most common malignant endocrine carcinoma (1, 2). With the rapid advancement of molecular and radiological technologies, the diagnostic accuracy on thyroid cancer has been improved (3). Papillary thyroid cancer (PTC), accounting for 85% to 90% of all thyroid carcinomas, has been increasing in incidence in recent years (4, 5), especially for papillary thyroid microcarcinoma (6). Central lymph node metastasis (CLNM) occurs frequently in PTC, with a prevalence that could be as high as 40% to 90% (7). It was reported that patients with CLNM might be more likely to have distant metastasis and poor survival than those without CLNM (8). Thus, central lymph node dissection (CLND) is required for these patients. However, considering operative complications such as laryngeal nerve paralysis and hypocalcemia caused by the removal of CLND, it is still controversial whether CLND should be performed in all PTC patients. There are also some patients with microscopic and undetectable CLNM who are hard to evaluate by preoperative examination (9), though the significant difference of prognoses among micrometastatic PTC patients who is typically resected with prophylactic CLND appears minimal (10), but whether micrometastases could cause recurrence or distant metastasis remains unclear. Therefore, it is clinically significance to identify patients with a high risk for CLNM before surgery.

Furthermore, central compartment of lymph nodes seems to be the first station of nodal metastasis among thyroid cancer (11). The current approaches to evaluating lymph status before operation mainly included ultrasonography (US) and invasive fine needle aspiration (FNA), though with limited sensitivity (12, 13). There is still lack of more accurate method for identifying the risk of cervical lymph node metastasis. Developing new diagnostic tools for predicting cervical lymph node status is highly necessary.

Machine learning (ML) is a novel computer-based method for data analysis that has been widely applied in clinical medicine (14). ML can find more interactions between variables and outcomes by learning from dataset patterns than conventional statistical methods such as multinomial naïve Bayes (MNB) (15). Since very few studies have developed ML-based predictive models for thyroid cancer, this study aims to construct multiple ML-based models for the preoperative prediction of CLNM and identify risk factors associated with CLNM in patients with PTC.

## Materials and Methods

### Patients

This retrospective study was approved by the Ethics Committee of Peking Union Medical College Hospital (PUMCH), and written informed consent was obtained from all patients. A total of 1103 patients who underwent initial thyroid resection in PUMCH between January 2018 and December 2019 were enrolled in this study. All patients had PTC proven by final pathology. The exclusion criteria were as follows: 1) other types of thyroid tumors; 2) undergoing any chemotherapy or radiotherapy for thyroid malignancy before surgery; 3) no CLND; and 4) incomplete clinical information.

### Surgical Approach

For unilateral lobe PTC, lobectomy plus isthmusectomy with ipsilateral CLND was performed. For bilateral PTC or PTC in the isthmus, total thyroidectomy with bilateral CLND was performed. If lateral lymph node metastasis was suspected by preoperative ultrasound or proved by fine-needle aspiration cytology, lateral lymph node dissection was also performed. The dissection of the central compartment was considered level VI and included the pretracheal, paratracheal, and prelaryngeal lymph nodes. The lateral lymph nodes involve levels II, III, IV, and V. All surgical specimens were identified through intraoperative frozen section and postoperative paraffin section examination by pathological experts from PUMCH.

### Clinical Characteristics and Ultrasonographical Features

The following clinical characteristics were retrospectively collected for analysis: age, sex, body mass index (BMI), mean arterial pressure (MAP), fasting blood glucose (FBG) and thyroid function test results. All data were recorded before operation. Thyroid function tests were performed within one month before the operation at our hospital and included assessments of triiodothyronine (T3), tetraiodothyronine (T4), free T3 (FT3), free T4 (FT4), thyroid stimulating hormone (TSH), thyroid peroxidase antibody (TPO-Ab) and thyroglobulin antibody (TG-Ab).

All patients underwent cervical US within one month before surgery at our hospital with Phillips IU 22 (Philips Healthcare, Eindhoven, Netherlands), GE Logiq 9 (GE Healthcare, Milwaukee, WI, USA) devices equipped with 5 to 12MHz linear-array transducer (Thermal Index in soft tissue=0.1, Mechanical Index=0.06). There were only few patients that also underwent cervical computed tomography (CT) and FNA, which were performed if necessary, and thus the findings of CT and FNA were not included in this study. Based on US, the following features were recorded: tumor size, tumor location, hypoechogenicity, multiple nodules, bilateral nodules, microcalcification, irregular shape, unclear margin and capsular invasion. Tumor size was defined as the maximal tumor diameter for unifocal cases and as the maximal diameter of the largest tumor for multifocal cases (16, 17). Tumor location was divided into two areas (left or right lobe, and isthmus). Classification of internal echogenicity was made as hypoechogenicity (totally solid) or non-hypoechogenicity (including mixed cystic, solid and iso-echoic nodules). The presence of multiple nodules was confirmed when there were other nodules (benign or malignant) in the thyroid in addition to the primary tumor. On a special occasion, when the additional nodules located in the contralateral lobe of the primary tumor, they were called bilateral nodules. Microcalcifications were defined as punctate echogenic foci ≤ 1 mm inside tumors. Classification of tumor shape was made as regular or irregular. Unclear margin was confirmed when the tumor nodules’ margin could not be well defined under US. Capsular invasion was defined as the disruption of the perithyroidal echogenic line between the thyroid capsule and the tumor. In addition, the metastatic status of cervical lymph nodes (LNs) on US was also included, which was based on multiple characteristics of lymph nodes, including size, shape, margin, cortex, echogenicity, echotexture, microcalcification, necrosis, hilar echogenicity and vascularity (18). For cases with multifocal thyroid tumors, some features such as hypoechogenicity, microcalcification, irregular shape, unclear margin and capsular invasion were identified if they were observed in any one of the tumors. All of the US features above were assessed by sonographers at our hospital with more than 10 years of experience in analyzing US image of thyroid cancer.

### Development of ML-Based Models

A total of 22 variables involving clinical characteristics and US features (**Table 2**) were used to develop ML-based models for the preoperative prediction of CLMN. Seven algorithms were applied in this study, including six representative supervised ML algorithms [random forest classifier (RFC), artificial neural network (ANN), decision tree (DT), gradient boosting decision tree (GBDT), extreme gradient boosting (XGBoost) and adaptive boosting (AdaBoost)] (19–24) and the conventional algorithms (MNB). Most of the algorithms are inexplicable except DT and MNB, in which the function between variables and the outcome cannot be visible to users. The ANN algorithm is a widely parallel inter-connected network composed of adaptable simple units, which can simulate the interaction of the biological nervous system with real-world objects. The DT algorithm divides a difficult prediction problem into two or more simpler subsets like branches of the tree, and thus into different sub-problems structurally. RFC is a more advanced algorithm based on DT, which can used for both regression and classification. GBDT, XGBoost and AdaBoost fall into one kind of important ML algorithms called ensemble learning. It can improve the generalization ability of the classifiers by training multiple classifiers and then combining them to achieve better prediction performance. Moreover, to construct more reliable models, all continuous variables underwent preprocessing for z-score normalization, except for MNB, in which min-max normalization is preferred (25).

The predictive performance of these models was evaluated by the area under the receiver operating characteristic (ROC) curve (AUC). In addition, decision curve analysis (DCA) was used to assess the clinical utility of these models. The predictive performance of cervical US for CLNM was also evaluated and compared with that of the models using a t-test.

### Validation Strategy and Feature Selection

To minimize the adverse effect of overfitting, a common problem in ML algorithms, 5-fold cross-validation and feature selection were performed (26, 27). A classifier-specific importance evaluator was applied to identify the optimal variables for each model (28). A ranked list of variables was generated for each model, and all of the variables were compared to determine their predictive importance for CLNM. The AUCs of different numbers of variables were also calculated to find the optimal dimension (number of variables) of each model.

### Statistical Analysis

Univariate and multivariate analysis (LR forward) was performed using IBM SPSS 25.0 (SPSS Inc; Chicago, IL, USA). The development and validation of ML-based models were performed using Python programming language (version 3.7, Python Software Foundation). Student’s t-test was used to compare models’ predictive performance (AUC). The normality of quantitative data was tested by the Shapiro-Wilk test. Non-normally distributed data are expressed as the median with interquartile range (IQR). Normally distributed quantitative parameters were compared by Student’s t-test, while non-normally distributed parameters were compared by the Mann-Whitney U test. For categorical data, Pearson’s chi square test was applied. A P value < 0.05 was considered statistically significant.

## Results

### Demographic Characteristics

As shown in **Table 1**, this retrospective study cohort consisted of 297 (26.9%) males and 806 (73.1%) females, with a median age of 41 (IQR: 33–51) years. Of all patients, the median BMI, MAP, FBG and tumor size were 24.33 (IQR: 21.97–26.67), 93 mmHg (IQR: 86–102), 5.1 mmol/L (IQR: 4.8–5.5), and 1.0 cm (IQR: 0.7–1.5), respectively. There were 560 (50.8%) patients with tumors ≤ 1.0 cm, 385 (34.9%) patients with tumors 1.0 to 2.0 cm and 158 (14.3%) patients with tumors ≥ 2.0 cm. The presence of multiple nodules was observed in 660 (59.8%) cases, and bilateral nodules were observed in 542 (49.1%) cases. A total of 612 (55.5%) patients were proven to have positive central LNs by postoperative pathology. There were 439 (39.8%) patients who underwent lobectomy plus isthmusectomy with ipsilateral CLND and 664 (60.2%) who underwent total thyroidectomy with bilateral CLND. The median number of harvested central LNs was 7 (IQR: 5–11), while that of positive central LNs was 3 (IQR: 1–5).

**Table 1:** Demographic characteristics of the patients.

Clinicopathological characteristics	Data
All patients, n (%)	1103 (100.0)
Age, years^†^	41 (33–51)
Sex, n (%)	
Male	297 (26.9)
Female	806 (73.1)
BMI^†^	24.33 (21.97–26.67)
MAP, mmHg^†^	93 (86–102)
FBG, mmol/L^†^	5.1 (4.8–5.5)
Tumor size, cm^†^	1.0 (0.7–1.5)
≤ 1.0	560 (50.8)
1.0–2.0	385 (34.9)
≥ 2.0	158 (14.3)
Multiple nodules, n (%)	
Yes	660 (59.8)
No	443 (40.2)
Bilateral nodules, n (%)	
Yes	542 (49.1)
No	561 (50.9)
Central lymph node metastasis	
Positive	612 (55.5)
Negative	491 (44.5)
Surgical resection, n (%)	
Lobectomy plus isthmusectomy with ipsilateral CLND	439 (39.8)
Total thyroidectomy with bilateral CLND	664 (60.2)
No. of harvested central lymph nodes^†^	7 (5–11)
No. of positive central lymph nodes^†^	3 (1–5)

^†^Continuous variables were expressed as median with interquartile range.

BMI, body mass index; MAP, mean arterial pressure; FBG, fasting blood glucose; CLND, central lymph node dissection.

### Univariate and Multivariate Analyses of Variables

Univariate analysis (**Table 2**) showed that CLNM was significantly associated with age, sex, FBG, FT3, FT4, TPO-Ab and US features such as tumor size, microcalcification, irregular shape and suspected LNs (P<0.05). Then, variables with P<0.05 in the univariate analysis were selected for multivariate analysis using LR forward stepwise selection. The results showed that age (OR=0.959, 95% CI: 0.948–0.971, P<0.001), male sex (vs. female, OR=1.527, 95% CI: 1.132–2.059, P=0.006), TPO-Ab (OR=0.998, 95% CI: 0.997–0.999, P=0.003) and US features such as tumor size (OR=1.234, 95% CI: 1.058–1.439, P=0.007), microcalcification (OR=1.911, 95% CI: 1.461–2.500, P<0.001) and suspected LNs (OR=3.268, 95% CI: 2.401–4.448, P<0.001) were independent risk factors for CLNM (**Table 3**).

**Table 2:** Univariate analysis of clinical characteristics and ultrasonography features related to central lymph node metastasis (CLMN).

Variable	Univariate analysis		
	CLMN (−)	CLMN (+)	P value
Age, years^†^	45 (37–53)	38 (31–47)	<0.001
Sex, n (%)			
Male	109 (22.2)	188 (30.7)	0.002
Female	382 (77.8)	424 (69.3)	
BMI^†^	24.46 (22.06–26.45)	24.22 (21.76–26.83)	0.764
MAP, mmHg^†^	94 (86–103)	93 (86–101)	0.295
FBG, mmol/L^†^	5.2 (4.9–5.6)	5.1 (4.8–5.5)	0.004
FT3, pg/ml^†^	3.06 (2.82–3.28)	3.15 (2.91–3.36)	<0.001
FT4, ng/dl^†^	1.21 (1.01–1.31)	1.22 (1.13–1.33)	0.048
T3, pg/ml^†^	1.04 (0.94–1.14)	1.04 (0.95–1.17)	0.247
T4, ng/dl^†^	7.90 (7.00–8.90)	7.63 (6.90–8.70)	0.090
TSH, μIU/ml^†^	1.72 (1.20–2.64)	1.76 (1.22–2.62)	0.941
TG-Ab, IU/ml^†^	13.69 (10.43–83.94)	13.17 (10.30–74.00)	0.467
TPO-Ab, IU/ml^†^	14.91 (12.04–23.84)	13.90 (11.47–19.67)	0.027
**Ultrasonography features**			
Tumor size, cm^†^	0.9 (0.7–1.3)	1.2 (0.9–1.7)	<0.001
Tumor location, n (%)			
Left or right lobe	471 (95.9)	590 (96.4)	0.680
Isthmus	20 (4.1)	22 (3.6)	
Hypoechogenicity, n (%)			
Yes	438 (89.2)	554 (90.5)	0.470
No	53 (10.8)	58 (9.5)	
Multiple nodules, n (%)			
Yes	306 (62.3)	354 (57.8)	0.132
No	185 (37.7)	258 (42.2)	
Bilateral nodules, n (%)			
Yes	253 (51.5)	289 (47.2)	0.155
No	238 (48.5)	323 (52.8)	
Microcalcification, n (%)			
Present	252 (51.3)	442 (72.2)	<0.001
Absent	239 (48.7)	170 (27.8)	
Irregular shape, n (%)			
Yes	328 (66.8)	452 (73.9)	0.011
No	163 (33.2)	160 (26.1)	
Unclear margin, n (%)			
Yes	336 (68.4)	439 (71.7)	0.233
No	155 (31.6)	173 (28.3)	
Capsular invasion, n (%)			
Yes	38 (7.7)	61 (10.0)	0.198
No	453 (92.3)	551 (90.0)	
Suspected LNs, n (%)			
Present	81 (16.5)	251 (41.0)	<0.001
Absent	410 (83.5)	361 (59.0)	

^†^Continuous variables were expressed as median with interquartile range.

BMI, body mass index; MAP, mean arterial pressure; FBG, fasting blood glucose; T3, triiodothyronine; T4, tetraiodothyronine; FT3, free T3; FT4, free T4; TSH, thyroid stimulating hormone; TPO-Ab, thyroid peroxidase antibody; TG-Ab, thyroglobulin antibody; LNs, lymph nodes.

**Table 3:** Multivariate analysis of clinical characteristics and ultrasonography features related to central lymph node metastasis (CLMN).

Variable	OR	95% CI	P value
Age	0.959	0.948–0.971	<0.001
Sex			
Male	1.527	1.132–2.059	0.006
Female	Reference	–	–
TPO-Ab	0.998	0.997–0.999	0.003
Tumor size	1.234	1.058–1.439	0.007
Microcalcification	1.911	1.461–2.500	<0.001
Suspected LNs	3.268	2.401–4.448	<0.001

TPO-Ab, thyroid peroxidase antibody; LNs, lymph nodes.

### Predictive Performance and Clinical Usefulness of ML-Based Models

**Figure 1:**
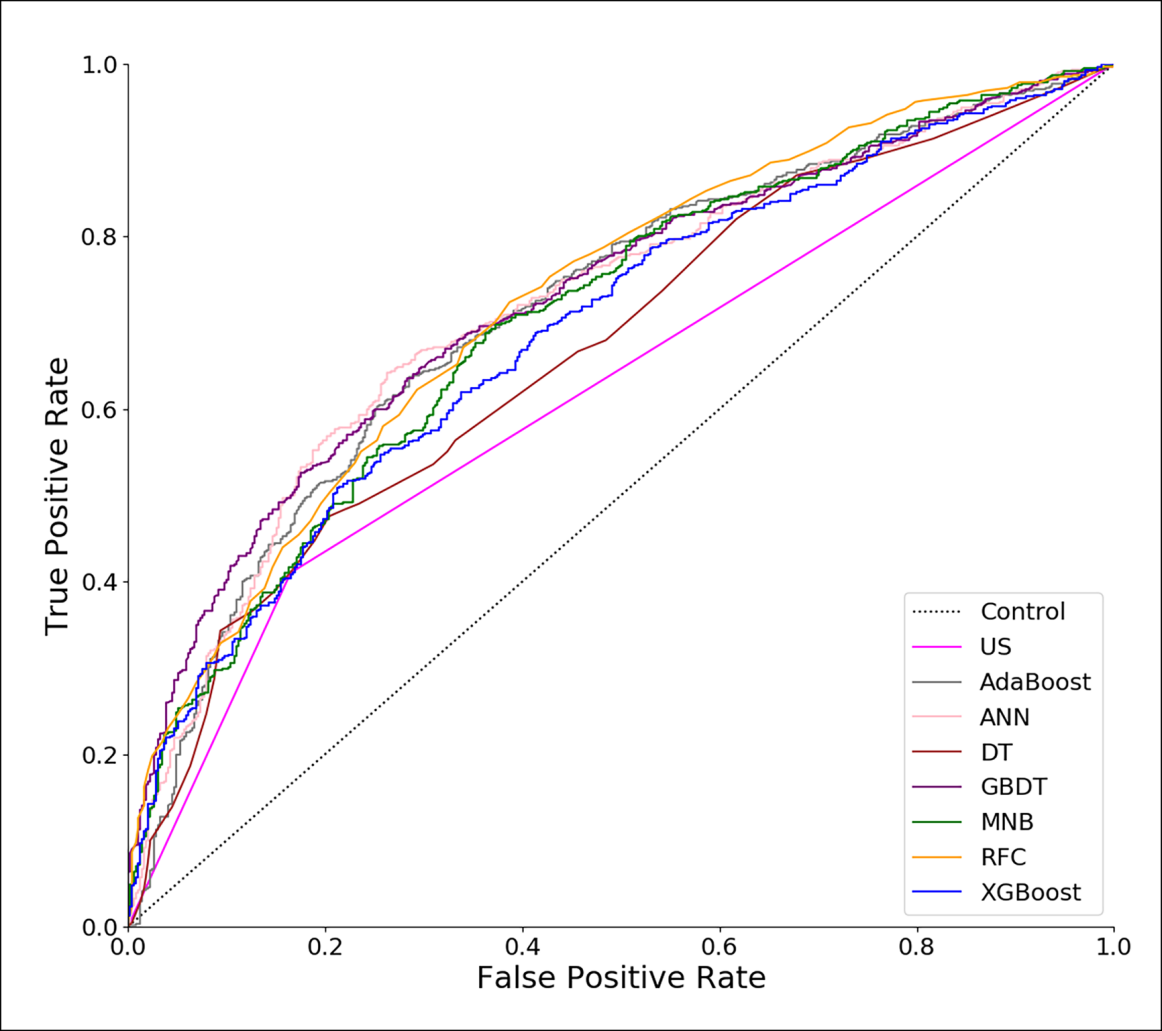
Receiver operating characteristic (ROC) curves of predictive models based on machine learning algorithms. US, ultrasonography; GBDT, gradient boosting decision tree; RFC, random forest classifier; AdaBoost, adaptive boosting; ANN, artificial neural network; MNB, multinomial naïve Bayes; XGBoost, extreme gradient boosting; DT, decision tree.

Using all 22 variables, predictive models for CLNM were developed based on 7 algorithms. The predictive performance of the models is shown in **Figure 1** and **Table 4**. All models we developed performed significantly better than US (AUC=0.623, SD=0.017, P<0.05). The AUC values of ML models were higher than the conventional MNB except DT and XGBoost, though the differences were not significant (P>0.05).

**Table 4:** Predictive performance of models and ultrasonography alone and their optimal number of dimensions (number of variables).

Model	AUC			No. of optimal dimension	Sensitivity	Specificity
	Mean	SD	P value^†^			
GBDT	0.731	0.015	<0.001	7	63.6%	71.7%
RFC	0.730	0.015	<0.001	12	72.4%	61.3%
AdaBoost	0.721	0.015	<0.001	8	63.7%	71.5%
ANN	0.718	0.015	<0.001	5	64.2%	73.7%
MNB	0.717	0.016	<0.001	5	69.8%	62.7%
XGBoost	0.690	0.016	0.002	14	51.0%	78.6%
DT	0.680	0.016	0.008	3	47.5%	79.6%
US	0.623	0.017	–	1	41.0%	83.5%

^†^P values were obtained when compared with US. Sensitivity and specificity were confirmed according to maximal Youden’s index.

AUC, area under the curve; SD, standard deviation; GBDT, gradient boosting decision tree; RFC, random forest classifier; AdaBoost, adaptive boosting; ANN, artificial neural network; MNB, multinomial naïve Bayes; XGBoost, extreme gradient boosting; DT, decision tree; US, ultrasonography.

**Figure 2:**
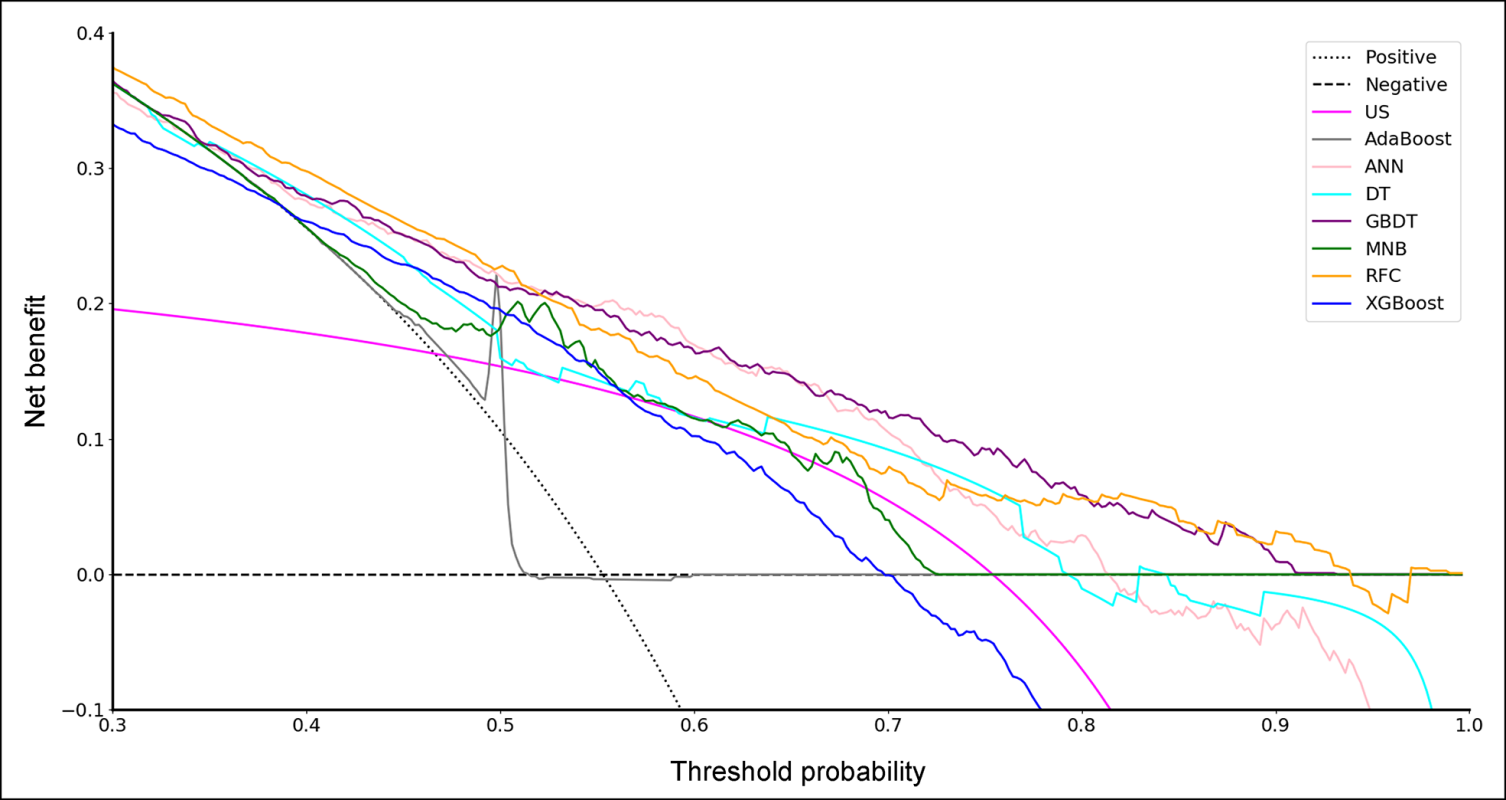
Decision curve for predictive models based on machine learning algorithms. US, ultrasonography; GBDT, gradient boosting decision tree; RFC, random forest classifier; AdaBoost, adaptive boosting; ANN, artificial neural network; MNB, multinomial naïve Bayes; XGBoost, extreme gradient boosting; DT, decision tree.

To evaluate the clinical utility of these models, DCA was performed (**Figure 2**). According to the incidence of CLNM among patients with PTC, the reasonable range of thresholds was set as 0.4 to 0.9. Almost at the entire range, all ML-based models showed higher net benefits than the two extreme lines (treat-none and treat-all) except AdaBoost. It was noteworthy that three ML-based model, GBDT, ANN and RFC, always performed better than US and other models. There were sharply decreases at the threshold range of 0.7 to 0.9 for ANN and 0.6 to 0.8 for RFC, but the GBDT model remained a stably-high net benefit across almost the entire reasonable range of threshold probabilities.

### Variable Importance

**Figure 3:**
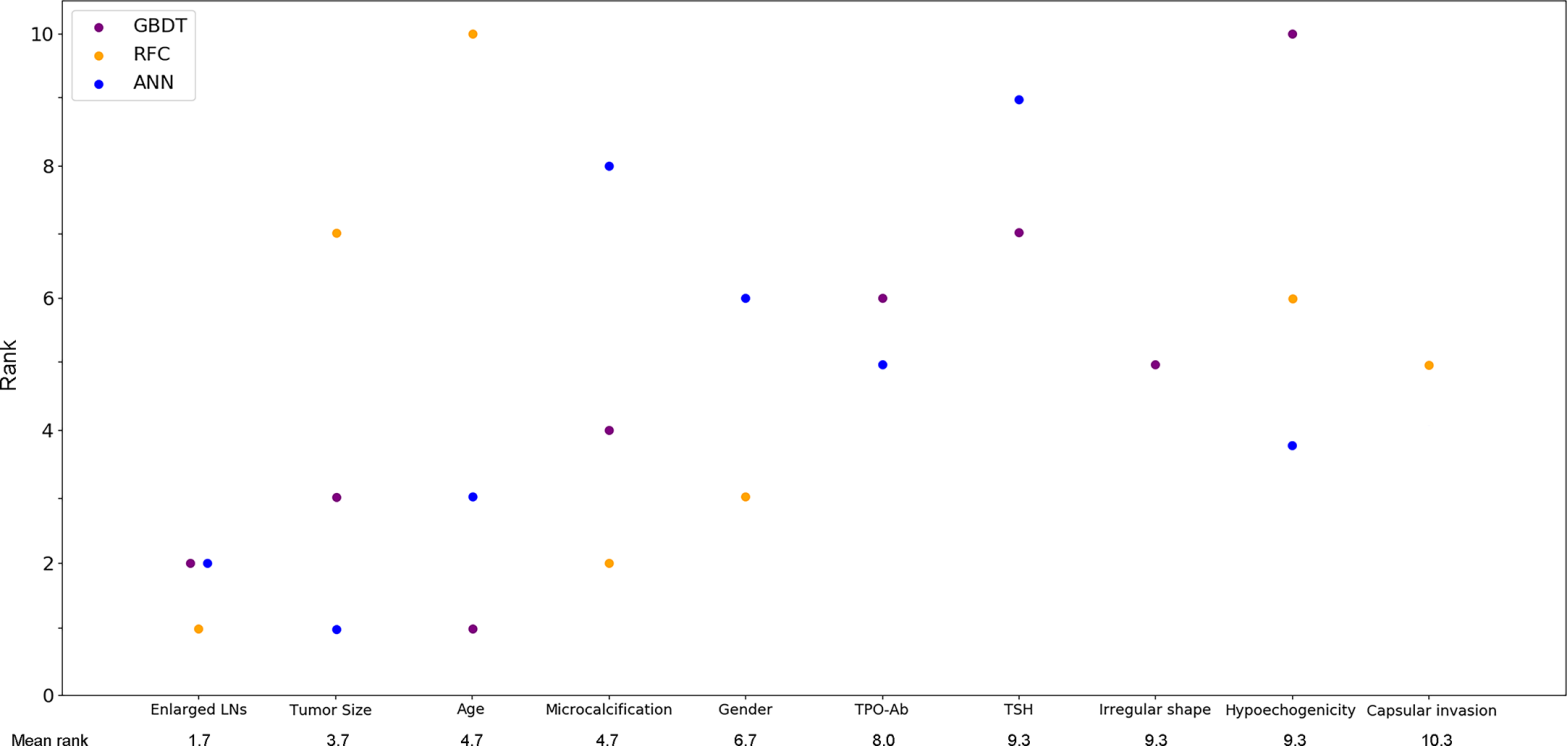
Ranks of the top 10 variables for the prediction of central lymph node metastasis. Variables were ranked using a classifier-specific importance evaluator based on machine learning algorithms. The variables are ordered according to the mean ranking of three potential models, which were GBDT, RFC and ANN. A lower rank represents more predictive importance. For example, age was ranked 1st, 3rd, and 10th in GBDT, ANN, and RFC, respectively. LNs, lymph nodes; Micro-Cal, microcalcification; TPO-Ab, thyroid peroxidase antibody; TSH, thyroid stimulating hormone; Ir. shape, irregular shape; Cap. invasion, capsular invasion; Hypo-echo, hypoechogenicity; GBDT, gradient boosting decision tree; RFC, random forest classifier; ANN, artificial neural network.

With favorable AUCs and clinical benefits according to the DCA, GBDT, RFC and ANN were selected to be the models with the most potential for predicting CLNM in PTC patients. By the feature selection approach, the top 10 variables were ranked based on their predictive importance in each potential model (**Figure 3**). The variables were arranged in order of mean ranking: suspected LNs, tumor size, age, microcalcification, gender, TPO-Ab, TSH, irregular shape, hypoechogenicity, and capsular invasion. The ranks of each variable in different models were described in **Supplementary Table 1**.

**Figure 4:**
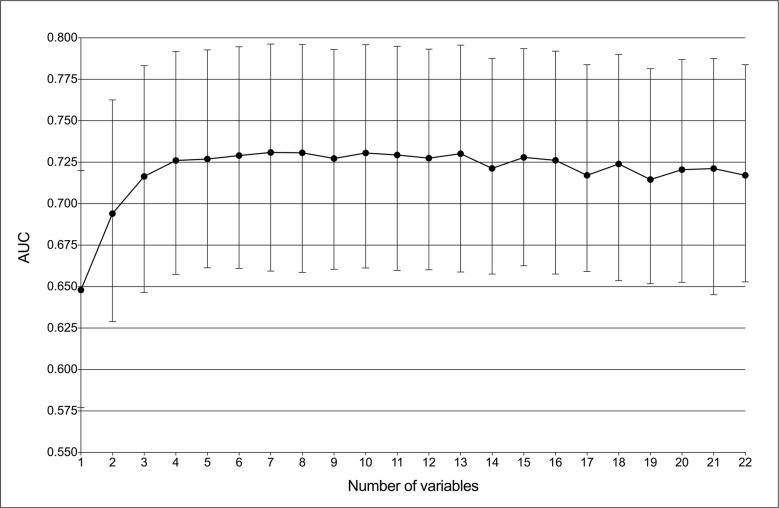
Predictive performance of the gradient boosting decision tree (GBDT) model with different numbers of variables. The AUC was the highest (0.731) with seven variables.

GBDT was identified as the best predictive model in this study because of its best performance in both ROC curve (**Figure 1**) and decision curve (**Figure 2**). The AUCs of GBDT reached the highest when 7 variables were introduced (**Figure 4**). These 7 variables were as follows: age, suspected LNs, tumor size, microcalcification, irregular shape, TPO-Ab and TSH.

## Discussion

Currently, the prevalence of PTC has shown rapid growth as US has been widely used for cervical examination. Despite the fact that patients with PTC have a 10-year survival rate of more than 90% (29), lymph node metastasis occurs very frequently. The central compartment is regarded as the first metastatic station, whose metastatic incidence could reach up to 90% (7). Previous studies have shown that CLNM was significantly associated with local recurrence and survival (8, 30, 31). Then, prophylactic CLND was proposed, but this procedure would be irrelevant for patients without nodal metastasis and even cause a higher incidence of complications. Thus far, whether to perform CLND for PTC patients without preoperative and intraoperative suspected lymph node metastasis remains controversial. There are no reliable predictive models for CLNM in PTC patients. Therefore, in addition to univariate and multivariate analysis, we developed multiple models for the prediction of CLNM by ML algorithms and compared these models with US. ROC analysis and DCA were used to assess these models’ predictive performance and clinical utility, respectively. Then, potential models were confirmed to identify risk factors for CLNM by feature selection.

In this study, a retrospective cohort of 1103 PTC patients was reviewed. A total of 22 variables including clinical characteristics and US features were used to predict CLNM. The predictive performance of all models was significantly better than that of US (**Table 4**). The three excellent models, including GBDT, RFC and ANN, performed better in both the ROC analysis and DCA than the other models (**Table 4**, **Figures 1** and **2**).

Despite the invisible connection between variables and outcomes in most ML-based models, the predictive importance of the variables in each potential model was obtained by using the classifier-specific evaluator (**Figure 3**). Thus, the top ten variables were considered to be the most important risk factors for CLNM: suspected LNs, tumor size, age, microcalcification, gender, TPO-Ab, TSH, irregular shape, hypoechogenicity and capsular invasion.

Currently, the definitive diagnosis of CLNM mainly depends on postoperative pathology. The preoperative risk factors for CLNM in PTC patients remain unclear. Our study suggested that CLNM had a significant tendency to appear in young patients. The optimal cut-off age was 40 years old (AUC=0.648; sensitivity: 0.68; specificity: 0.56), which is similar to previous studies that reported age < 45 years was a risk factor for CLNM (16, 17, 32, 33). In addition, a sex predisposition was also observed in our study. We found that males were associated with a higher incidence of CLNM, which was also supported by previous reports (16, 17, 32–35). Overall, male patients < 40 years old might be considered a high-risk population for CLNM and should be evaluated carefully when choosing surgical procedures. In addition, TPO-Ab was also identified as an independent risk factor by multiple analysis and ML algorithms. Actually, several studies have reported the association between nodal metastasis and chronic lymphocytic thyroiditis (CLT), which can demonstrate an increased serum level of TPO-Ab. A meta-analysis from Lee et al. concluded that CLT occurred more in PTC patients but might indicated no lymph node metastasis. However, Antonio et al. found the coexistence of CLT among PTC patients was associated with more risk of nodal metastasis. On the other hand, very few studies focused on the association between TPO-Ab and CLNM. In our study, we found that a reduced serum TPO-Ab level might indicate a high risk for CLNM, but this association still requires further confirmation of future prospective studies. The TSH value was not significantly different between CLNM (+) and CLNM (–) patients in our study or previous studies (16), but this variable was ranked 7th according to the mean rankings of three potential models (**Figure 3**). TSH was also involved in the GBDT model with a rank of 7. This might also indicate the superiority of ML algorithms on data mining and reveal that variables having a P value > 0.5 in the use of conventional statistical methods should not be totally overlooked.

Previously, the sensitivity of US in predicting LNM was reported to be as low as 41.3% to 61.0%, although US is most commonly used for the assessment of cervical LNs (36–38). Other imaging examinations, such as CT, performed slightly better in terms of sensitivity than US, but the difference was not significant (37, 38). In our study, all patients underwent preoperative US, which showed that 332 (30.1%) patients possessed suspected LNs (AUC=0.623; sensitivity: 0.41; specificity: 0.83). Moreover, the presence of suspected LNs was also identified as the most predictive risk factor for CLNM based on ML models. Thus, the previous suggestion that all patients with suspected lymph nodes on US should undergo CLND is reasonable, but US alone is still not enough to predict CLNM status. Tumor size on US images is an important indicator of tumor growth. It has been reported that large tumor size was an independent risk factor for CLNM, but the cut-off values are inconsistent. Liu and Ahn et al. thought that tumors > 1.0 cm were a risk factor for CLNM (16, 17), while Zhou et al. suggested a tumor size threshold > 0.7 cm (39). Our study also indicated that tumor size was an independent risk factor for CLNM (**Table 3**), and this variable ranked third in mean ranking of ML models (**Figure 3**). The optimal cut-off tumor size was calculated to be > 1.1 cm (AUC=0.634; sensitivity: 0.59; specificity: 0.37), which is almost consistent with that in previous reports (16, 17). In addition, microcalcification on US images also indicated CLNM. Eun et al. classified calcifications inside thyroid carcinoma into four types: microcalcification, macrocalcification, rim calcification and non-calcification (40). Many studies have reported that the presence of microcalcification was significantly associated with a higher incidence of cervical LNM (16, 40, 41). It is thought that the formation of microcalcifications is caused by the rapid proliferation of cancer cells (42). Therefore, PTC patients with the presence of microcalcification on US images should be evaluated more carefully before surgery. Furthermore, irregular shape, capsular invasion and hypoechogenicity might be potential risk factors for CLNM. Tumors with these US features deserve more attention.

In addition to the clinical indications, there were some methodological innovations in this study. First, this is the first study to develop ML-based models for the prediction of CLNM in PTC patients. By incorporating clinical characteristics and US features, these ML-based models showed excellent predictive performance and clinical utility by ROC analysis and DCA. Second, in addition to conventional multivariate analysis, using feature selection approach, we identified risk factors for CLNM by mean ranking three well-selected ML-based models. The mean ranks of these variables indicated their predictive importance. Third, our study identified the best ML-based model for the prediction of CLNM in PTC patients, which was the GBDT model with 7 variables. In the future, an online application of the GBDT model should be developed based on the clinical characteristics and US features to allow surgeons and patients in other hospitals to benefit from this study.

Several limitations were needed to be noted. First, this is a retrospective study in which data bias might be unavoidable. A prospective cohort should be used to construct an ML-based model for further evaluation. Second, more than 50% of the tumors included were microcarcinomas (≤ 1 cm). Actually, many other centers do not biopsy or operate on thyroid nodules less than 1 cm, which limited the reproducibility of this study.

## Conclusions

Using ML algorithms, it is feasible to incorporate clinical characteristics and US features to predict CLNM in PTC patients. All ML-based models performed better than US in the prediction of CLNM. The GBDT model with 7 variables was identified as the best model according to ROC analysis and DCA. Based on multivariate analysis and feature selection, younger age, male sex, low serum TPO-Ab and US features such as suspected LNs, microcalcifications and tumor size > 1.1 cm were important risk factors for CLNM. ML algorithms can be useful for the prediction of lymph node metastasis in PTC.

## Data Availability Statement

The raw data supporting the conclusions of this article will be made available by the authors,without undue reservation.

## Ethics Statement

The studies involving human participants were reviewed and approved by Ethics Committee of Peking Union Medical College Hospital. The patients/participants provided their written informed consent to participate in this study.

## Author Contributions

Conceptualization: ZL, XX, YW, and KR. Methodology: YW, CH, and. JL. Formal analysis: YW, KR, CH, JL, YC, and LG. Investigation: YW, KR, and LG. Writing—original draft preparation: YW. Writing—review and editing: YW, KR, ZL, and XX. Supervision: YW and XX. All authors contributed to the article and approved the submitted version.

## Conflict of Interest

The authors declare that the research was conducted in the absence of any commercial or financial relationships that could be construed as a potential conflict of interest.
