# Supplementary material for: Machine Learning Algorithms for the Prediction of Central Lymph Node Metastasis in Patients With Papillary Thyroid Cancer
Source: Front Endocrinol (Lausanne). 2020 Oct 21;11:577537. doi: 10.3389/fendo.2020.577537 (PMC7609926; doi:10.3389/fendo.2020.577537)
Supplement: Supplementary file 1 [file Table_1.docx]

**Table S1. Ranks of 22 variables at each model.**

| Variable | ADB | ANN | DT | GBDT | MNB | RFC | XGB |
| --- | --- | --- | --- | --- | --- | --- | --- |
| Suspected LNs | 2 | 2 | 1 | 2 | 1 | 1 | 1 |
| Age | 1 | 3 | 13 | 1 | 4 | 10 | 3 |
| Tumor size | 3 | 1 | 16 | 3 | 10 | 7 | 10 |
| Microcalcification | 7 | 8 | 2 | 4 | 2 | 2 | 2 |
| Gender | 5 | 6 | 3 | 11 | 15 | 3 | 4 |
| TPO-Ab | 11 | 5 | 22 | 6 | 3 | 13 | 17 |
| TSH | 21 | 9 | 14 | 7 | 14 | 12 | 21 |
| Irregular shape | 12 | 12 | 7 | 5 | 22 | 14 | 12 |
| Capsular invasion | 6 | 13 | 8 | 10 | 5 | 5 | 9 |
| Hypoechogenicity | 17 | 4 | 9 | 18 | 21 | 6 | 7 |
| Bilateral nodules | 13 | 20 | 5 | 9 | 18 | 4 | 5 |
| FT4 | 4 | 21 | 10 | 8 | 11 | 9 | 11 |
| T4 | 14 | 10 | 20 | 14 | 9 | 18 | 14 |
| Multiple nodules | 9 | 11 | 4 | 21 | 20 | 11 | 13 |
| FBG | 22 | 22 | 15 | 13 | 6 | 8 | 19 |
| FT3 | 10 | 15 | 21 | 16 | 12 | 15 | 16 |
| MAP | 20 | 14 | 19 | 17 | 17 | 16 | 22 |
| BMI | 16 | 18 | 12 | 12 | 13 | 19 | 15 |
| TG-Ab | 19 | 7 | 17 | 22 | 7 | 21 | 18 |
| Tumor location | 8 | 17 | 18 | 20 | 16 | 17 | 6 |
| Unclear margin | 15 | 19 | 6 | 15 | 19 | 20 | 8 |
| T3 | 18 | 16 | 11 | 19 | 8 | 22 | 20 |

AUC: area under the receiver operating characteristic curve; AdaBoost: adaptive boosting; ANN: artificial neural network; DT: decision tree; GBDT: gradient boosting decision tree; MNB: multinomial Naïve Bayes; RFC: random forest classifier; XGBoost: extreme gradient boosting; BMI: body mass index; MAP: mean arterial pressure; FBG: fasting blood glucose; T3: triiodothyronine; T4: tetraiodothyronine; FT3: free T3; FT4: free T4; TSH: thyroid stimulating hormone; TPO-Ab: thyroid peroxidase antibody; TG-Ab: thyroglobulin antibody; LNs: lymph nodes.
